# Surgical Inflammation Alters Immune Response to Intraoperative Photodynamic Therapy

**DOI:** 10.1158/2767-9764.CRC-22-0494

**Published:** 2023-09-11

**Authors:** Richard W. Davis, Astero Klampatsa, Gwendolyn M. Cramer, Michele M. Kim, Joann M. Miller, Min Yuan, Cassandra Houser, Emma Snyder, Mary Putt, Sergei A. Vinogradov, Steven M. Albelda, Keith A. Cengel, Theresa M. Busch

**Affiliations:** 1 Department of Radiation Oncology, Perelman School of Medicine, University of Pennsylvania, Philadelphia, Pennsylvania.; 2 Department of Medicine, Perelman School of Medicine, University of Pennsylvania, Philadelphia, Pennsylvania.; 3 Department of Biostatistics, Perelman School of Medicine, University of Pennsylvania, Philadelphia, Pennsylvania.; 4 Department of Biochemistry and Biophysics, Perelman School of Medicine, University of Pennsylvania, Philadelphia, Pennsylvania.

## Abstract

Surgical cytoreduction for patients with malignant pleural mesothelioma (MPM) is used for selected patients as a part of multi-modality management strategy. Our group has previously described the clinical use of photodynamic therapy (PDT), a form of non-ionizing radiation, as an intraoperative therapy option for MPM. Although necessary for the removal of bulk disease, the effects of surgery on residual MPM burden are not understood. In this bedside-to-bench study, Photofrin-based PDT introduced the possibility of achieving a long-term response in murine models of MPM tumors that were surgically debulked by 60% to 90%. Thus, the addition of PDT provided curative potential after an incomplete resection. Despite this success, we postulated that surgical induction of inflammation may mitigate the comprehensive response of residual disease to further therapy. Utilizing a previously validated tumor incision (TI) model, we demonstrated that the introduction of surgical incisions had no effect on acute cytotoxicity by PDT. However, we found that surgically induced inflammation limited the generation of antitumor immunity by PDT. Compared with PDT alone, when TI preceded PDT of mouse tumors, splenocytes and/or CD8^+^ T cells from the treated mice transferred less antitumor immunity to recipient animals. These results demonstrate that addition of PDT to surgical cytoreduction significantly improves long-term response compared with cytoreduction alone, but at the same time, the inflammation induced by surgery may limit the antitumor immunity generated by PDT. These data inform future potential approaches aimed at blocking surgically induced immunosuppression that might improve the outcomes of intraoperative combined modality treatment.

**Significance:**

Although mesothelioma is difficult to treat, we have shown that combining surgery with a form of radiation, photodynamic therapy, may help people with mesothelioma live longer. In this study, we demonstrate in mice that this regimen could be further improved by addressing the inflammation induced as a by-product of surgery.

## Introduction

Malignant pleural mesothelioma (MPM) is an aggressive thoracic cancer for which surgical cytoreduction as a part of a multi-modality management strategy can improve patient outcomes (1). To treat residual disease after surgical cytoreduction, our group has investigated the use of intraoperative photodynamic therapy (PDT), which employs a systemically delivered photosensitizer (Photofrin) in conjunction with illumination of the chest cavity (2). PDT kills tumor cells through the generation of reactive oxygen species (3), destroys tumor vasculature (4–6), and induces both innate and adaptive immune responses (7–9). However, unlike classical radiation, PDT restricts dosing healthy tissue due to the limitations of light penetration. Encouragingly, a median survival of 31.7 months was achieved in a retrospective analysis of a phase I trial in which Photofrin-mediated PDT was delivered intraoperatively to patients with late-stage (stage III/IV) MPM after macroscopic complete resection (2).

However, it is well recognized that surgery can induce an immunosuppressive state (10–15). Necrosis induced by surgery suppresses Th1 cytokines (such as IL2, IL12, and IFNγ) and shifts immunity toward a Th2 response through increases in IL6, IL8, IL10, and TNFα (16). The result is an increased presence of regulatory T cells (Treg), myeloid-derived suppressor cells (MDSC), and M2 macrophages alongside dysfunctional CD8^+^ T cells (16–18).

We postulated that this effect would work to counteract PDT induction of antitumor immunity. To mimic the observed mechanical and inflammatory stress of the surgical setting in a controlled laboratory environment, we have previously developed a tumor incision (TI) murine model that mimics the inflammatory cytokine signaling during surgery in human patients with mesothelioma (19). In this model, a tumor is incised, but not removed or otherwise manipulated. This causes the induction of surgical injury with its accompanying changes in the local tumor microenvironment, as well as the systemic changes associated with surgery (e.g., secretion of IL6) without changing the tumor size, thus allowing much more controlled comparisons with tumors not subjected to TI.

The objective of this study was to examine the interaction between surgical inflammation and PDT using the TI model. Results will inform strategies to maximize treatment outcomes in patients with locally advanced MPM treated with surgical cytoreduction.

## Materials and Methods

### Surgical Resection and TI Models

Studies were conducted using tumors propagated from the murine mesothelioma cells lines AB12 (RRID:CVCL_4405) and AE17ova.meso (ovalbumin and mesothelin transfected from parent RRID:CVCL_LJ85, herein shortened to AE17o; refs. 20, 21). All cell lines were generously provided by Steven Albelda. Cell authentication and *Mycoplasma* testing were conducted for all cell lines by IDEXX BioAnalytics. Cell authentication was performed using a mouse short tandem repeat profile and interspecies contamination test. This test involved a species-specific PCR evaluation. *Mycoplasma* testing was performed using a PCR evaluation for detection of *Mycoplasma pulmonis* and *Mycoplasma sp.* Animal studies were approved by the University of Pennsylvania Institutional Animal Care and Use Committee and animal facilities are accredited by the American Association for the Accreditation of Laboratory Animal Care. Partial surgical resection and TI models were performed as outlined previously (19). Tumors were propagated by the intradermal injection of 1 × 10^6^ AB12 or AE17o cells into the right flank of female 6–8 weeks old BALB/c mice (RRID:ISMR_JAX:000651) or C57BL/6 mice (RRID:ISMR_JAX:000664), respectively (Charles River Laboratories) and monitored until they reached the indicated volumes using the following formula: π/6 * (tumor length) * (tumor width)^2^. Treatment groups were randomly assigned. Mice were provided analgesia via subcutaneous injection of buprenorphine (ZooPharm) and anesthetized via inhalation of isoflurane in medical air (VetEquip anesthesia machine). In a sterile field, an incision was generated parallel to tumor growth and a skin flap was generated to expose the tumor. For partial surgical cytoreduction, the longest length of the exposed tumor was measured, and the tumor volume was excised at 30%, 60%, or 90% of the measured value. For TI, the tumor was incised one-half its depth along the longest axis without subsequent removal of tumor burden. In both cases, skin flaps were replaced and closed via sutures and mice were provided subcutaneous fluids. TI experiments were repeated in male BALB/c mice bearing AB12 tumors.

### PDT

Mice were injected with 5 mg/kg Photofrin (Pinnacle Biologics) via tail vein, followed 1 day later with light delivery at 632 nm through microlens-tipped fibers. Light was produced with a Ceralas Biolitec laser, measured via a Labmaster power meter (Coherent) and delivered over a 1.1-cm spot to an irradiance of 75 mW/cm^2^ and a total fluence of 135 J/cm^2^. To provide immobilization, mice were anesthetized via inhalation of isoflurane in medical air (VetEquip anesthesia machine). In combinations of PDT with TI, the tumor incision was introduced at 4 hours prior to PDT, except for a single study in which TI was introduced immediately after PDT.

### Tumor Optical Properties

Three optical parameters relevant for PDT are: (i) the average absorption coefficient (μ_a_), representing the optical absorption of the tissue; (ii) the reduced scattering coefficient (μ’_s_), representing the light scattering properties of the tissue at the near-infrared wavelengths (λ = 600–800 nm); and (iii) effective attenuation coefficient (μ_eff_), representing the total effective attenuation, which is calculated by μ_eff_ = √(3μ_a_μ’_s_). These were measured using a custom made, multi-fiber spectroscopic contact probe, described elsewhere (22). Briefly, the probe is comprised of two source fibers connected to a white light source to collect diffuse reflectance and a 405 nm laser source for fluorescence excitation. The detector fibers were connected to a multi-channel charge coupled device (CCD) spectroscopy system. Tumor optical properties were measured before and after treatment.

### Phosphorescence Lifetime Oximetry of Tumor Oxygenation

One day prior to exposure to TI, mice were injected with 40 μmol/L Oxyphor G4 (PdG4; ref. 23) by intratumoral injection (0.02 mL at the indicated concentration). The next day, the baseline level of oxygenation was measured as described previously (24). Briefly, mice were anesthetized (isoflurane) and tumor oxygenation was measured via using a fiber-optic phosphorometer (Oxyled, Oxygen Enterprises Ltd.). The probe was excited at λ = 635 nm and the phosphorescence was detected by an avalanche photodiode. Phosphorescence produced by the pulse was captured by the quartz optical fiber and delivered to a CCD camera for quantification and calculation. Phosphorescence measurements were taken in triplicate at three distinct points that were removed from or along the incision site. Thirty minutes after the baseline measurement, mice were subjected to TI or left uninjured, followed by oxygen measurements at 2, 4, and 6 hours.

### Spectrophotometric Assessment of Photofrin Levels

Tissue (50 mg) was solubilized in 0.5 mL of Solvable (Packard) overnight (20 ± 2 hours) prior to mixing with an equal volume of distilled water. Spectrofluorometry was performed with a Fluoromax-4 spectrofluorometer (Horiba) at 405 nm excitation and 627 nm emission. Photofrin concentration was calculated on the basis of the increase in fluorescence resulting from the addition of a known amount of Photofrin to each sample after its initial reading.

### *In Vivo*/*In Vitro* Clonogenic Assay

As described previously (25), tumors were excised at indicated timepoints, weighed, minced, enzymatically digested and plated for colony formation. Data are expressed as clonogenic cells per gram of tumor (calculated as cells per gram of tumor times the ratio of the number of colonies counted to the number plated).

### Histologic Analysis

Tumors were removed 24 hours after treatment with PDT in the presence or absence of TI, placed inside TRUFLOW tissue cassettes (Thermo Fisher Scientific), and submerged in 10% formalin overnight. After fixation, tissues were embedded in paraffin, sectioned, stained with hematoxylin and eosin (H&E), and assessed by an independent pathologist through the Comparative Pathology Core at the University of Pennsylvania School of Veterinary Medicine.

### Enzyme-linked Immunoassay

Tumors were excised and prepared for immunoassay using a previously described protocol (19). Briefly, tumors were frozen in a slurry of dry ice and 70% ethanol prior to homogenization and submission to three freeze-thaw cycles. Protein concentration was measured via bicinchoninic acid assay and proteins were resuspended to a concentration of 1.0 to 1.2 mg/mL. Samples were plated in duplicate on IL6 and mKC ELISA plates (R&D Systems) at dilutions of 1:1 and 1:2 as per manufacturer's instructions. Final values were adjusted for protein concentration and dilution factors.

### Winn Assay

Winn assays were performed by subcutaneously injecting mice with mixes of tumor cells and isolated splenocytes or CD8^+^ T cells from previously treated animals. Recipient mice were injected with 2 × 10^6^ AB12 cells (BALB/c model) or 2 × 10^6^ AE17o cells (C57BL/6 model) mixed with either whole splenocytes or purified CD8^+^ T cells isolated from spleens of donor mice. The donor tumor-bearing mice were sacrificed 2 days after treatments (PDT, TI, TI/PDT; *n* = 10 per donor group), their spleens were harvested and the splenocytes or CD8^+^ T cells were isolated. In experiments for which whole splenocytes were used, sample splenocyte single-cell suspensions were subjected to flow cytometric staining and analyzed to determine CD8^+^ T-cell frequencies; inoculum was adjusted to a splenocyte CD8:tumor cell ratio of 3:1. In the experiments for which spleen CD8^+^ T cells were used, CD8^+^ T cells were isolated from single-cell suspensions via magnetic labeling and subsequently separated using the murine CD8α^+^ T-cell Isolation Kit and LS columns (both from Miltenyi Biotec). Samples from isolated cells were tested for CD8^+^ T-cell purity via flow cytometry. Inoculum for recipient animals was adjusted to a splenocyte CD8:tumor cell ratio of 3:1. All flow cytometric testing was done as described in Flow Cytometry section, using antibodies against CD3 (17A2, BioLegend), CD4 (GK1.5, BioLegend), and CD8 (53-6.7, BioLegend). As controls, splenocytes or CD8^+^ cells from nontreated tumor-bearing mice, as well as naïve mice, were also isolated and coinjected with tumor cells. The tumor cell/lymphocyte mixtures were injected into five recipient mice per treatment condition and tumor growth was measured daily with calipers.

### Flow Cytometry

Tumors, spleens, and tumor-draining lymph nodes (TDLNs) were analyzed by flow cytometry where indicated. Briefly, spleens and lymph nodes were mechanically sheared using the plunger of a 1 mL syringe, with digests passed through a 40 μm cell strainer in the presence of RPMI1640 medium containing 10% heat-inactivated FBS and 1% penicillin/streptomycin. When needed, red blood cells were lysed via resuspension in Ammonium-Chloride-Potassium (ACK) Lysing buffer. Tumors were enzymatically digested and processed using a validated protocol (26). The resulting pellets were resuspended in PBS and stained with Live/Dead fluorescent reactive dye (1:400 in PBS, Invitrogen) and mouse Fc block (1:200 in PBS, 2.4G2, BD Biosciences) for 10 minutes at 4°C, followed by cell surface marker antibodies for 45 minutes, 4°C. Antibodies included: CD8a (53-6.7), CD4 (GK1.5), CD3 (17A2), CD45 (30.F11), Ly6G (1A8), CD11b (M1/70), Ly6C (HK1.4), and PD-L1 (B7-H1; all from BioLegend). For transcription factor FOXP3 intracellular staining, cells stained for surface markers were washed, fixed with Fix/Perm (eBioscience) for 1 hour at 4°C, washed twice and stained in the presence of Perm/Wash (eBioscience) for 45 minutes at 4°C. Flow cytometric analysis was performed on a BD LSR Fortessa with FACS Diva Software (BD Biosciences) and analyzed using FlowJo 10.2. Gating schemes are presented alongside individual experiments.

### Statistical analysis

Time-to-event analyses were used to assess differences in tumor response defined by regrowth to a specified tumor volume, taking into account censoring for animals that did not regrow their tumors by a prespecified time [i.e., those with a complete response (CR)]. Kaplan–Meier plots were created and differences in regrowth rates were primarily assessed using log-rank tests. For the CD8^+^ antibody tumor response, a parametric survival model with a Weibull distribution and a Wald test was used to assess differences between groups.

Tumor optical properties were compared using parametric, paired *t* tests between pre- and post-TI values. For the comparison of multiple groups, differences in the distributions were assessed using a Kruskal–Wallis test; if the global test reached statistical significance, individual groups were compared using Dunn method to address multiple comparisons.

Growth-delay analysis was used for Winn assay experiments; the outcome was set as the number of days needed to achieve a volume reached by 50% of the control animals on day 7. The transfer of AB12 T cells was instead analyzed by one-way ANOVA with adjustment for multiple comparisons using Tukey approach for tumor volumes due to the lack of regrowth in tumors containing T cells transferred from control animals.

Analyses were done in R version 4.2.1 using the flexsurv library for the parametric survival analysis and PRISM V 9.0.0. The type I error rate was set to 0.05 and all hypothesis tests were two sided.

### Data Availability

The data generated in this study are available upon request from the corresponding author.

## Results

### Intraoperative PDT Introduces Curative Potential When Added to Incomplete Resection

**FIGURE 1:**
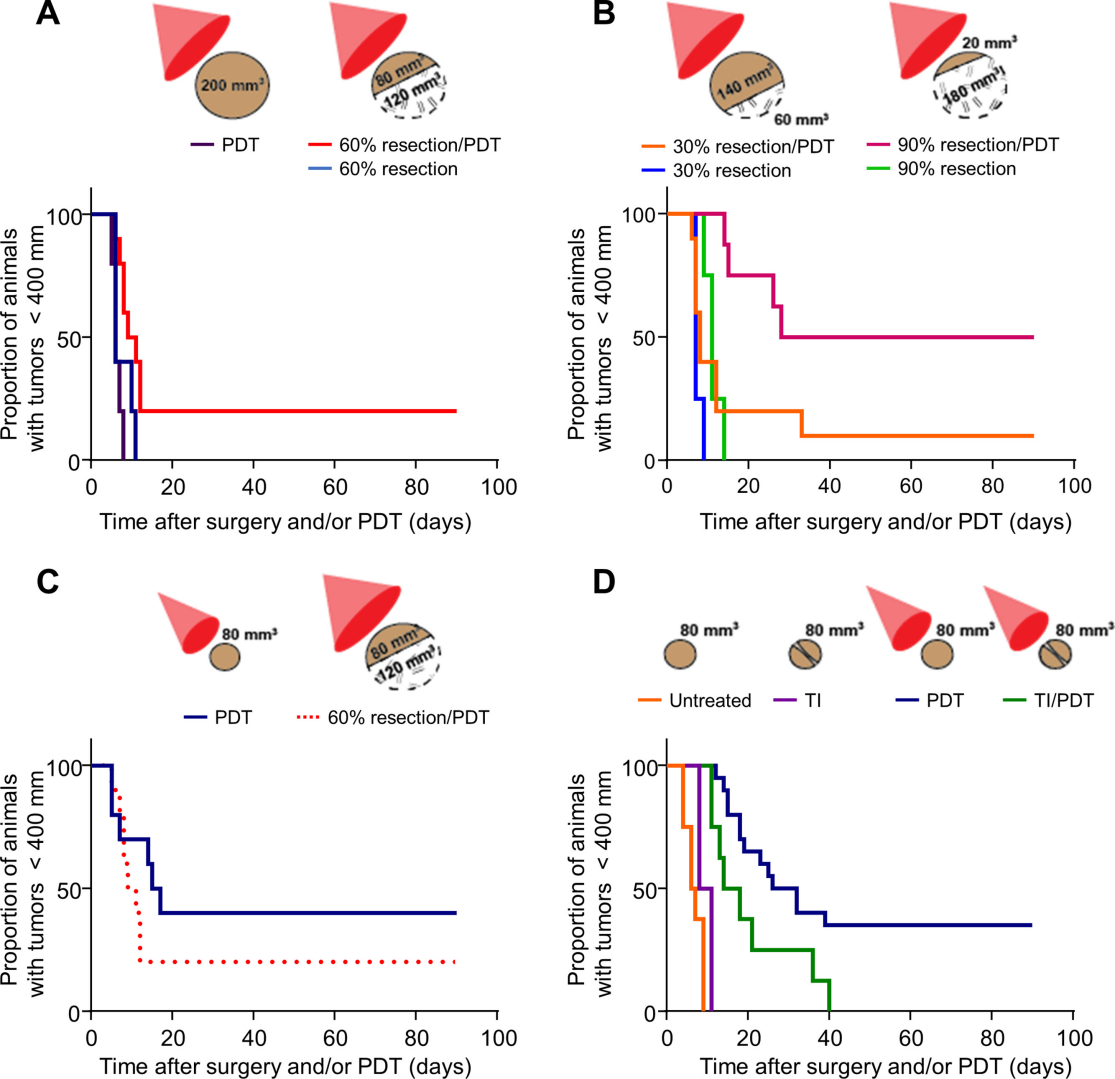
Surgical resection, although necessary, limits the achievable PDT response of residual disease. Murine mesothelioma AB12 cells were implanted in the flanks of female syngeneic mice. **A,** Mice with 200 mm^3^ tumors that were surgically debulked by 60% (to ∼80 mm^3^) prior to PDT (*n* = 10) achieved CRs (no regrowth after 90 days) and showed longer times to regrowth than mice whose tumors were treated with PDT alone (*n* = 5; *P* = 0.115) or tumors debulked by 60% alone (*n* = 5; *P* = 0.008). **B,** Mice bearing 200 mm^3^ tumors were surgically debulked by either 30% (*n* = 10) or 90% (*n* = 8) prior to treatment with PDT or in the absence of PDT (*n* = 4 each). For surgically debulked tumors treated with PDT, a larger extent of cytoreduction significantly increased the response rate (*P* = 0.014). **C,** CR rates to PDT were numerically higher in tumors that received PDT at 80 mm^3^ (*n* = 10) versus those that were grown to 200 mm^3^ and surgically debulked to 80 mm^3^ prior to PDT (*n* = 10, shown again from A). **D,** Tumors were exposed to TI by creating a skin flap and incision across the longest diameter. Mice administered TI prior to PDT (TI/PDT, *n* = 8) showed worse response rates than those treated with PDT alone (*n* = 20; *P* = 0.022).

Mice bearing large (200–300 mm^3^) AB12 tumors treated by partial surgical cytoreduction (down to 60% of the original tumor mass) regrew tumors on day 8; similarly, mice whose large tumors were treated by PDT alone regrew tumors on day 11. The addition of PDT to surgical cytoreduction yielded CRs (marked by no regrowth at 90 days) in 2 of 10 animals and a 30% resection produced CR in 1 of 10 mice (Fig. 1A and B, growth curves for individual mice are shown in Supplementary Fig. S1). PDT following more extensive resection produced better outcomes; PDT of large (200–300 mm^3^) murine tumors after 90% resection produced CR in 4 of 8 mice (Fig. 1B). Thus, the addition of PDT to a maximum achievable macroscopic resection is the best approach to achieve long-term tumor control.

### Independent of Cytoreduction, Surgical Intervention Can be Inhibitory

In diseases not amenable to complete resection, the above data demonstrate the benefit of combining PDT with macroscopic resection. However, given the known immunosuppressive effects of surgery, we hypothesized that the addition of surgery might inhibit the response to PDT. PDT applied to tumors resected from 200 to 80 mm^3^ achieved a CR in 2 of 10 mice (20%); PDT applied to tumors grown to an equivalent size of 80 mm^3^ achieved a CR in 4 of 10 mice (40%; Fig. 1C).

The partial cytoreduction approach introduces two major limitations in studying tumor regrowth. First, the tumor microenvironment of the residual tumor is mismatched to the 80 mm^3^ primary tumor. Second, the approach introduces human error in consistently reducing tumors to the same sizes within each subgroup. Therefore, we sought to utilize a model of surgical inflammation independent of cytoreduction; this model would allow tumors of a consistent size and microenvironment to enter the study at the time inflammation is induced. To assess the effect of surgery on the response to PDT independently of tumor resection, we used our TI model (19). Like our previous study, TI administration generated a significant, transient increase in IL6 levels in tumor (105 pg/mL 4 hours after TI vs. 30 pg/mL baseline, *P* = 0.015; Supplementary Fig. S2), as well as significant increases in tumor neutrophils (7.48% 4 hours after TI vs. 2.34% at baseline, *P* = 0.027; 6.12% 19 hours after TI, *P* = 0.003; Supplementary Fig. S2). Mice that received TI followed by PDT (TI/PDT) fared worse than those that received PDT in the absence of this surgical injury (Fig. 1D); specifically, CR of 80 mm^3^ tumors to PDT occurred in 7 of 20 mice (35%), whereas no CR was achieved when 80 mm^3^ tumors were injured by incision (TI) prior to PDT light delivery (PDT vs. TI/PDT, *P* = 0.022). Therefore, when resection was replaced with only the injury that it produces (i.e., TI), we recapitulated the observed inhibitory effect of surgery on PDT (see Fig. 1C).

Using the TI model, we further confirmed the inhibitory effect of TI on PDT for AB12 tumors propagated on male animals (Supplementary Fig. S3). The addition of TI prior to PDT resulted in significantly poorer tumor control in male mice (2/15 CR for PDT vs. 0/15 CR for TI/PDT, *P* = 0.040 for tumor regrowth rates), as was found in female animals.

To confirm that the effect of TI on PDT response was specific to its introduction prior to light delivery for PDT, tumor response studies were repeated with the change in sequence where TI was introduced immediately after PDT (PDT/TI; Supplementary Fig. S4). Unlike TI/PDT, PDT/TI produced a response that was indistinguishable from PDT alone [7/20 CR for PDT-treated (replotted from Fig. 1D) vs. 6/23 CR for PDT/TI-treated mice]. These data confirmed that TI was inhibitory only when introduced before PDT.

### Tumor Incision Does not Significantly Change the Acute Cytotoxicity of PDT

Possible mechanisms by which TI could impede PDT response include changes in the tumor environment due to mechanical damage. This led us to assess the effect of TI on tumor optical properties and tumor oxygenation, because both of these factors could change PDT efficacy by altering its production of reactive oxygen species.

**FIGURE 2:**
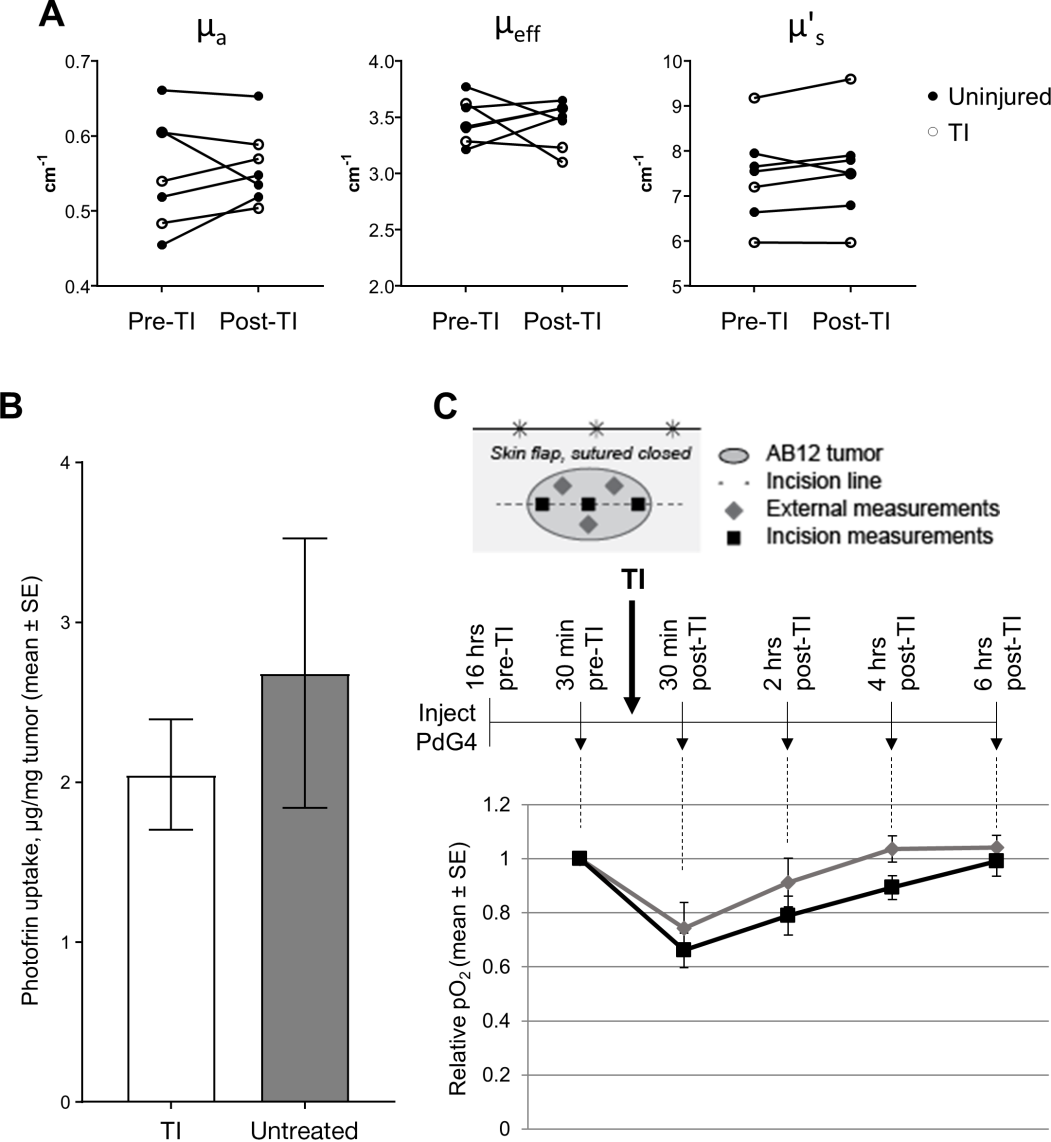
Optical properties, Photofrin content, and oxygenation are similar at the time of illumination in PDT- and TI/PDT-treated tumors. **A,** Tumor optical properties were measured prior to and immediately after TI exposure. After TI, tumors showed no difference in the mean of the average absorption (μ_a_, *P* = 0.690), effective coefficient (μ_eff_, *P* = 0.826), or reduced scattering coefficient (μ_s_’, *P* = 0.283). *n* = 6–7 mice per group. **B,** Spectrofluorometric analysis of tumors showed equivalent Photofrin uptake in TI-exposed and untreated tumors. *n* = 4–5 mice per group. **C,** Tumor oxygenation was measured using phosphorescence lifetime imaging via intratumoral injection of PdG4. Oxygenation levels are plotted relative to the pre-TI level in each mouse. The resulting curve demonstrated a transient decrease in tumor oxygenation 30 minutes after administration of TI which returned to a mean of 100% or 89.4% of the pre-TI level external to the incision and along the incision, respectively. *n* = 4 mice.

Tumor optical properties were measured prior to and at 4 hours after introduction of TI, that is, the time of light delivery for PDT. AB12 tumors showed no TI-based differences in mean values for μ_a_, μ_eff_, or μ_s_’ (Fig. 2A). Therefore, TI did not induce changes in tumor-averaged light propagation at the time of its delivery for PDT. We also confirmed that mechanical damage to the tumor, as well as the subsequent secretion of inflammatory cytokines such as IL6, did not affect the overall level of Photofrin in the tumor (Fig. 2B).

Next, we measured tumor oxygenation via the phosphorescent probe Oxyphor G4 (PdG4; ref. 23) at times before and after tumor incision. Transient hypoxia was detected at 30 minutes post-TI, which recovered to within 90% or greater of the pre-incision value by 4 hours after TI (Fig. 2C). Therefore, TI did not introduce significant hypoxia at the time of PDT.

**FIGURE 3:**
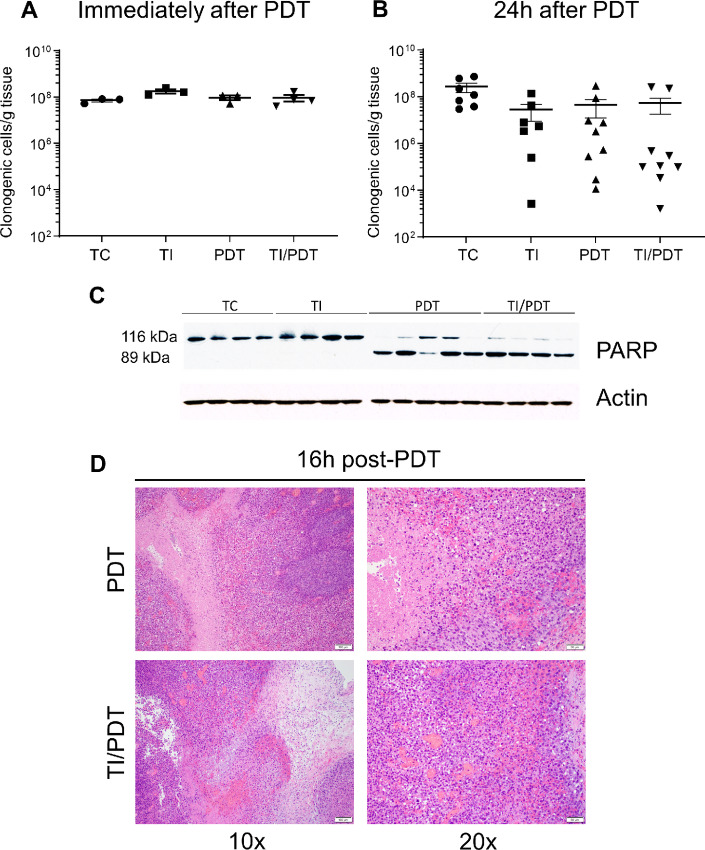
TI does not significantly impede direct PDT damage. Clonogenic analysis of tumor tissue immediately (**A**) or 24 hours (**B**) after PDT. At 24 hours after TI and/or PDT, reductions were observed in the median number of clonogenic cells per gram of tumor tissue compared with untreated controls (9.3-fold change for TI, 5.9-fold change for PDT, and 4.9-fold change for TI/PDT). Significance was only observed in the TI/PDT group versus untreated tumor control (TC, *P* = 0.0192). **C,** Immunoblot analysis of PARP cleavage 24 hours after PDT administration revealed no differences in TI-exposed tumors. *n* = 4 mice per group. **D,** Histologic samples taken from TI-, PDT-, or TI/PDT-treated tumors. H&E-stained sections are shown at 10X and 20X magnification. One representative image is shown from three repetitions showing similar extent of necrosis between PDT- and TI/PDT-treated tumors. *n* = 7–11 mice per group.

On the basis of these results, it would be expected that the short-term cytotoxic effects of PDT would not be affected by TI. Indeed, no difference was found in the clonogenic potential (clonogenic cells/g) of cells isolated from tumors immediately after PDT (Fig. 3A). Decreases in clonogenicity were observed 24 hours after TI and/or PDT (Fig. 3B); however, variability was observed in clonogenicity and was substantiated by analysis of PARP cleavage by Western blot analysis in PDT- and TI/PDT-treated tumors 24 hours after PDT administration (Fig. 3C). Histologic analyses of tumors at 16–18 hours after PDT or TI/PDT similarly detected the presence of severe regional or marked diffuse necrosis and severe inflammation consisting primarily of neutrophils at a cellular level (Fig. 3D). Notably, intertumor variability in the extent of this inflammation could contribute to the variability in the clonogenicity data because inflammatory cell infiltrate and fluid accumulation will alter tumor wet weight and thus values of clonogenic cells per gram. Collectively, these data show that TI did not alter the acute cytotoxic potential of PDT as measured through microenvironmental (light propagation or oxygenation) factors and molecular or clonogenic cell death. Moreover, the variable clonogenic cell death at 24 hours after PDT for the PDT and TI/PDT conditions, together with its similarity to the TI condition, points to minimal contributions from direct tumor cell death and acute vascular damage to the observed differences in antitumor efficacy for PDT in the presence or absence of TI.

### PDT Outcome is Dependent on Antitumor Immunity

**FIGURE 4:**
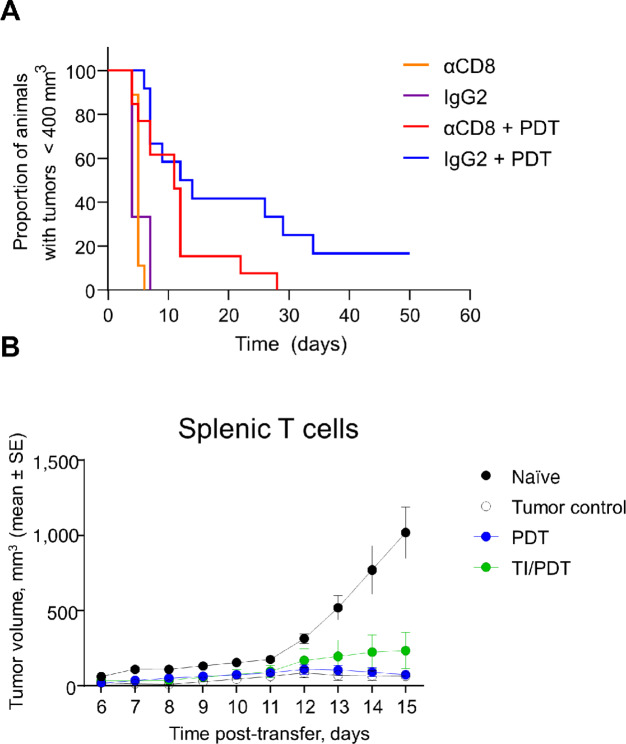
CD8^+^ T cells mediate a robust anti-PDT response and transfer antitumor activity. **A,** In mice bearing AB12 tumors, administration of CD8^+^-depleting antibodies prior to PDT (*n* = 13) impeded tumor response compared with mice treated with isotype control prior to PDT (*n* = 12; *P* < 0.001). The median time of tumor growth to the 400 mm^3^ endpoint was 4–5 days in mice treated with CD8^+^-depleting antibodies (*n* = 9) or isotype (*n* = 3) in the absence of PDT. **B,** T cells were isolated from the spleens of naïve mice, tumored mice, or mice whose tumors were treated with PDT or TI/PDT and transferred alongside AB12 cells into recipient mice. T cells isolated from the spleen of mice bearing tumors generated growth delays in recipient mice compared with the T cells isolated from the naïve group; this growth delay was maintained when the T cells were alternatively harvested from PDT or TI/PDT treated mice relative to the naïve group. *n* = 5 recipient mice per group.

Given limited evidence that TI altered light propagation for PDT or that the antitumor effects of PDT were mediated by either direct cell death or clonogenicity-altering acute vascular effects, we next considered whether TI alters immune responses to PDT. Antibody-mediated depletion of CD8^+^ T cells blunted the number of CRs to PDT (Fig. 4A). Compared with isotype alone, CD8^+^ antibody induced a 60% reduction in the risk of tumor regrowth after PDT. This suggests that a CD8^+^ response contributed to the beneficial effect of PDT after surgery. We hypothesized that CD8^+^ T cells isolated at 2 days after PDT would induce transferable antitumor immunity. To test this, CD8^+^ T cells were isolated from the spleens of differently treated mice and coinjected subcutaneously with tumor cells into new hosts with subsequent tumor growth measured (Winn assay). As shown in Fig. 4B, CD8^+^ T cells isolated from tumor-bearing animals, as well as those from tumor-bearing animals 2 days after treatment with PDT or TI/PDT, suppressed the growth of AB12 compared with tumors on mice cocultured with CD8^+^ T cells from animals without tumors.

Collectively, these data indicate that CD8^+^ T cells are required to obtain a CR to PDT of AB12 tumors, but PDT or TI/PDT did not discernably alter CD8^+^ T-cell immunity, perhaps because AB12 tumors themselves were highly effective in generating CD8^+^ T cells with antitumor immunity.

### Tumor Incision Suppresses PDT-generated Antitumor Immunity

To determine whether PDT and TI/PDT altered other aspects of the immune environment that might indirectly affect CD8^+^ T-cell activity, we performed another Winn assay using the whole spleen digest (splenocytes) mixed with tumor cells. In this way, the observed antitumor immunity reflected the activity of other immune cells in the spleen that could alter the action of CD8^+^ T cells.

**FIGURE 5:**
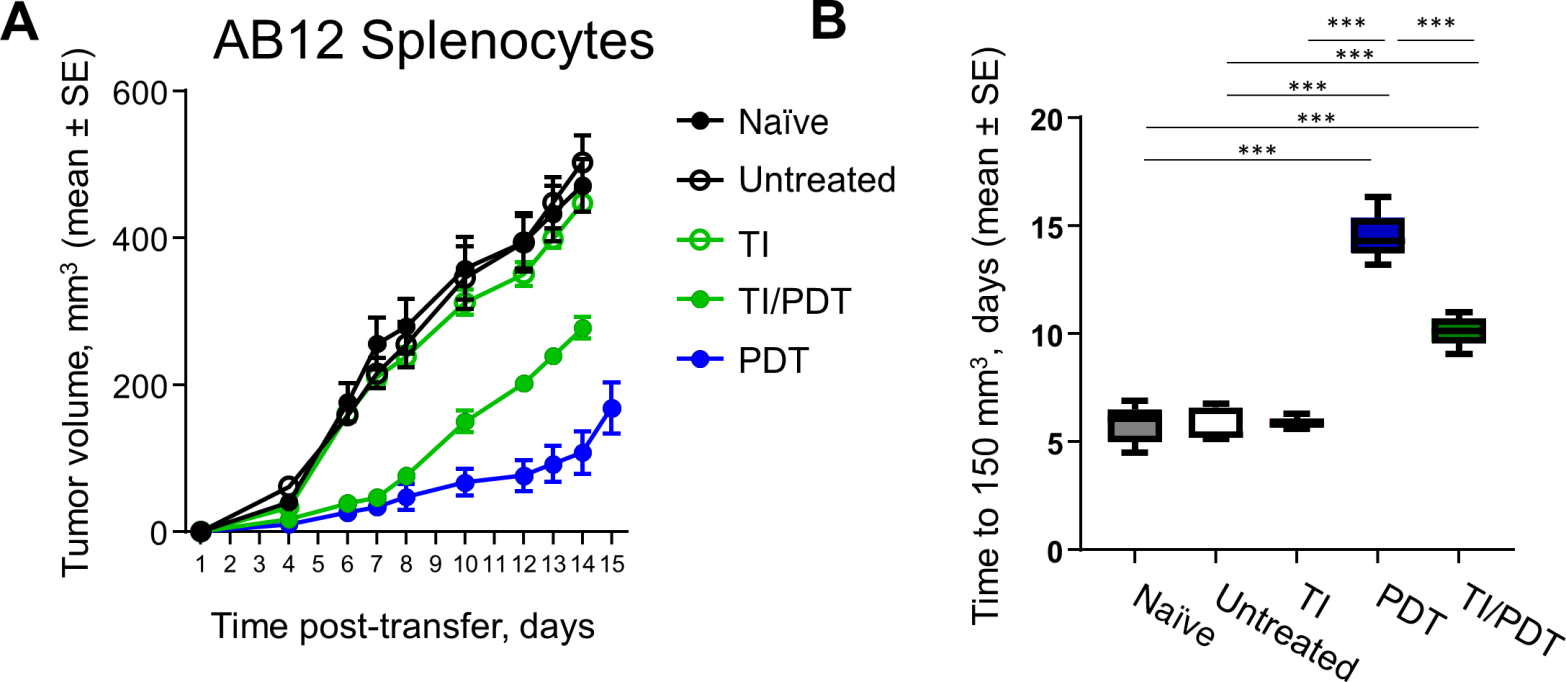
Splenocyte cells from TI/PDT treated mice transfer immunosuppression that limits PDT-induced antitumor immunity. Splenocytes isolated from the spleen of naïve mice, tumored mice, or tumored mice treated with TI, PDT, or TI/PDT were transferred alongside AB12 cells into recipient mice. Daily average tumor volume in recipient mice (**A**) and number of days for tumor growth to 150 mm^3^ (**B**) are shown. Splenocytes transferred from mice whose tumors were treated with PDT significantly delayed the growth of tumors. Compared with splenocytes isolated from mice receiving PDT alone, splenocytes transferred from mice whose tumors received TI prior to PDT permitted faster tumor growth in recipient mice. *n* = 5 recipient mice per group; *, *P* < 0.05; **, *P* < 0.01; ***, *P* < 0.001.

In contrast to the effects of adding CD8^+^ T cells alone (Fig. 4B), tumor growth was not blocked in untreated or TI treated mice (Fig. 5A), suggesting inhibitory cells (possibly MDSCs or CD4^+^ Tregs) were also present. Splenocytes from PDT-treated donor mice strongly inhibited tumor growth. Figure 5B further shows that transferring splenocytes from PDT-treated mice led to more significant growth delays for tumors in recipient mice than those transferred from donor mice bearing untreated tumors (*P* < 0.001). Preceding PDT by TI reduced the antitumor activity of splenocytes compared with those from mice treated with PDT alone; growth delays in recipient mice induced by transferring splenocytes from TI/PDT-treated mice were significantly less than those induced from PDT-treated mice (*P* < 0.001).

**FIGURE 6:**
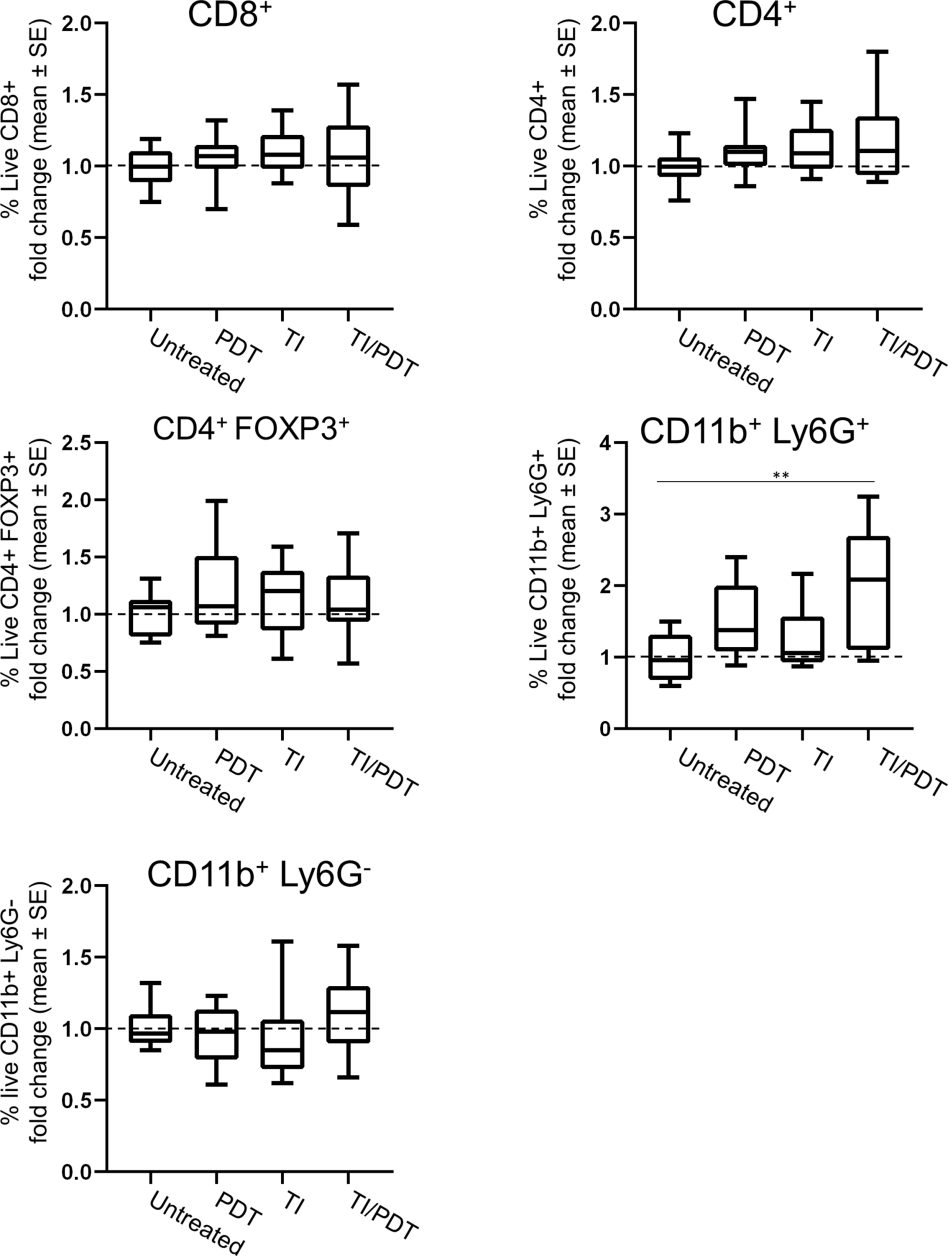
Immunophenotyping reveals increased granulocytes when TI precedes PDT. Flow cytometric analysis of splenocytes revealed no significant increase in CD8^^+^^ or CD4^+^ T cells at 2 days after administration of TI, PDT, or TI/PDT. Similarly, no variations were observed in CD4^+^FOXP3^+^ Tregs. Mean fold change of CD11b^+^Ly6G^+^ granulocytes in TI/PDT were significantly greater compared with untreated tumors; no significance was observed in tumors treated with PDT in the absence of TI. This was specific for Ly6G^+^ cells, as Ly6G^−^ myeloid cells were not affected by TI and/or PDT. *n* = 10 mice/group; *, *P* < 0.05; **, *P* < 0.01; ***, *P* < 0.001.

To inform results of the Winn Assay, immunophenotyping (using gating schemes in Supplementary Fig. S5) was performed at the timepoint of immune cell isolation from the donor mice. These data demonstrated that numbers of CD8^+^ T cells, CD4^+^ T cells, and Tregs (CD4^+^FOXP3^+^) in the spleens of mice that received PDT, TI, or TI/PDT did not change relative to their levels in untreated animals (Fig. 6). We further evaluated populations of CD11b^+^ Ly6G^+^ and CD11b^+^Ly6G^−^ cells, which would include MDSCs as either granulocytic MDSCs (CD11b^+^ Ly6G^+^) or monocytic MDSCs (CD11b^+^ Ly6G^−^). Levels of CD11b^+^ Ly6G^−^ cells were similar across all treatment conditions. The proportion of live cells in the spleen that were CD11b^+^ Ly6G^+^ was 3.79% in mice whose tumors were treated with PDT and 5.74% for mice whose tumors were treated with TI/PDT compared with 2.46% in mice whose tumors were untreated. The mean fold change in CD11b^+^ Ly6G^+^ levels was significantly greater in the spleen of mice whose tumors were treated with TI/PDT compared with untreated controls (*P* = 0.019). The mean fold change in CD11b^+^ Ly6G^+^ cells was also numerically greater for TI/PDT compared with PDT. Significance was not achieved between TI/PDT and PDT groups, perhaps owing to the variability observed in PDT-treated mice (i.e., 35% having the ideal scenario to achieve CR in Fig. 1D). Unlike in mice with TI/PDT-treated tumors, the mean fold change in CD11b^+^ Ly6G^+^ cells for mice with PDT-treated tumors was not significantly greater than those with untreated tumors.

### TI/PDT Generates a CD8^+^ T Cell–driven Response in Mesothelioma of C57BL/6 Mice

**FIGURE 7:**
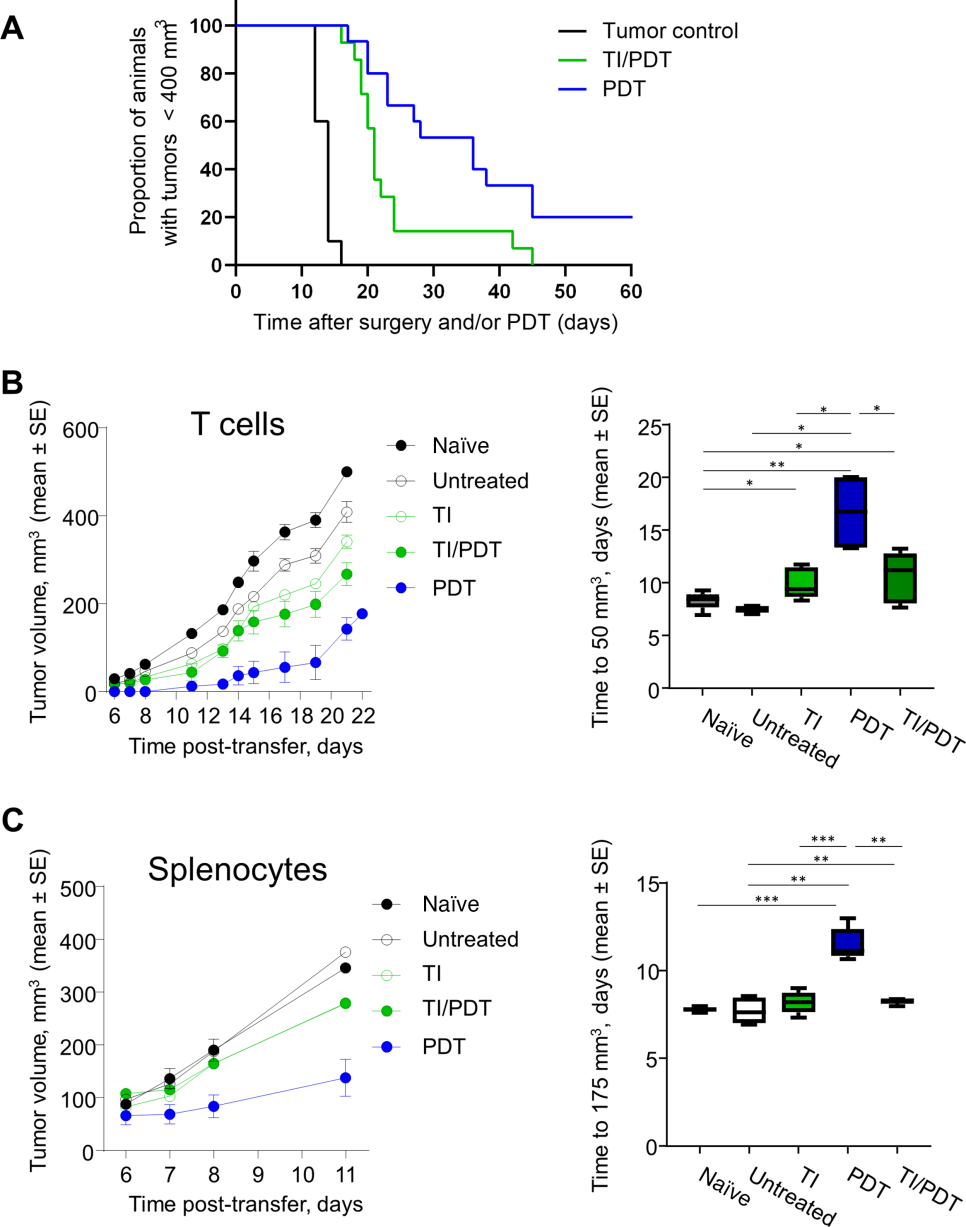
T cells and splenocyte cells from TI/PDT treated mice limit PDT-induced antitumor immunity in a C57BL/6 model of mesothelioma. Murine mesothelioma AE17o cells were implanted into the flank of C57BL/6 mice and grown to 80 mm^3^ prior to treating with TI and/or PDT. **A,** Tumor responses to PDT (*n* = 14) were impeded by exposure to TI prior to PDT (*n* = 15; *P* = 0.019 for PDT vs. TI/PDT). Transferability of antitumor immunity in AE17o tumors was assessed using CD8^+^ T cells isolated from splenocytes (**B**), or using whole splenocyte populations, from tumored, PDT-, or TI/PDT-treated mice (**C**). T cells or splenocytes were transferred alongside AE17o tumor cells to recipient mice. Preceding PDT with TI reduced the antitumor immunity encoded in CD8^+^ T cells (*P* = 0.0364 for TI/PDT vs. PDT) and whole splenocytes (*P* = 0.0017 for TI/PDT vs. PDT). Growth delay analysis for AE17o tumors represents growth to 50 mm^3^ for T cell and 175 mm^3^ for splenocyte transfer. *n* = 5 recipient mice/group; *, *P* < 0.05; **, *P* < 0.01; ***, *P* < 0.001.

A second murine mesothelioma cell line, AE17o, that grows in C57BL/6 mice (27) was used to confirm the immunosuppressive effects of TI/PDT observed in the AB12 model in BALB/c mice. Exposure of 80 mm^3^ AE17o flank tumors to TI 4 hours prior to administration of PDT reduced the overall survival compared with PDT (*P <* 0.001; Fig. 7A). Winn assays with purified splenic CD8^+^ T cells were performed (Fig. 7B). In this model, CD8^+^ T cells from untreated tumor-bearing animals had no significant effect on tumor growth compared with CD8^+^ T cells from naïve mice. This suggests that the AE17o tumors in C57BL/6 mice engender a weaker endogenous antitumor immune response than AB12 cells in BALB/c mice. Transferring CD8^+^ T cells from the spleens of PDT-treated mice led to a greater growth delay compared with those transferred from mice with untreated tumors (*P* = 0.019). Growth delays produced from CD8^+^ T cells transferred from TI/PDT-treated mice were significantly less than those transferred from PDT-treated mice (*P* = 0.036). CD8^+^ T cells taken from TI-treated mice did not significantly delay tumor growth relative to those taken from mice whose tumors were untreated (*P* = 0.071). These data indicate TI followed by PDT induces immunosuppression more directly on CD8^+^ T cells in the mesothelioma model in C57BL/6 mice than that observed in the mesothelioma model in BALB/c mice (see Fig. 4B).

We next conducted Winn assays using the transfer of whole splenocyte populations to include any immunosuppressive cells together with the CD8^+^ T cells (Fig. 7C). Similar to Winn assays with the whole spleen digest in the AB12 model, splenocytes donated from PDT-treated mice and cultured with AE17o cells generated significantly longer tumor delays compared with those donated from mice whose tumors were not treated (*P* = 0.001); the growth delay from TI/PDT-treated donors was significantly reduced compared with PDT-treated donors (*P =* 0.002).

## Discussion

Surgery is often used in combination with intraoperative adjuvant therapies in malignant mesothelioma, gynecologic cancers, soft-tissue tumors, and breast cancer (28). Because of the penetration limits of visible light, PDT is an excellent candidate for administration in combination with tumor cytoreduction in tumors with primarily superficial spread. However, common to these intraoperative applications is the potential for surgery to alter the action of subsequently delivered therapy, especially due to the temporal closeness of therapy with the tissue injury introduced by surgery. Such changes could include the effect of surgery on the physical characteristics of the remaining tumor and/or local or systemic immunologic changes. To optimize combination therapy, it is therefore essential to first understand which surgical-induced changes affect subsequent PDT and the mechanisms of such interactions.

To study this question, we undertook investigations in two murine models of mesothelioma. Long-term tumor control was achieved when tumor resection was combined with PDT (as is done clinically; Fig. 1B). Importantly, however, when we compared the efficacy of PDT on small tumors (that had not been resected) to tumors that had been resected to be the same size, we saw significantly more efficacy in the tumors that were not surgically manipulated (Fig. 1C). These results suggested that the process of surgery inhibited the efficacy of PDT.

Because of the complexities and variabilities of the effects of partial surgical removal of tumor, we used our previously published murine model of surgical injury (19) to study the mechanisms of the interactions between surgery and PDT in a more controlled setting. As we saw with actual surgical cytoreduction, adding a tumor incision to PDT significantly reduced therapeutic efficacy (Fig. 1D).

As others have found (29–31), induction of antitumor targeted CD8^+^ T cells was important to achieve long-term responses from PDT. We observed a loss of efficacy of PDT after CD8^+^ T-cell depletion in the AB12 model (Fig. 4A). We explored this issue more directly by studying the ability of CD8^+^ T cells purified from the spleens of tumor-bearing mice (with and without various treatments) to control tumor growth *in vivo* by injecting a mixture of immune cells and tumor cells (Winn assay), and we found that our two models had some interesting differences. At baseline, the AB12 tumor was extremely immunogenic, that is, CD8^+^ T cells from nontreated tumor-bearing mice had very strong antitumor activity. This activity was so strong that we were unable to detect any further augmentation by PDT (Fig. 4B). The AE17o tumor in C57BL/6 mice was comparatively non-immunogenic. In this model, the CD8^+^ T cells from nontreated tumor-bearing mice had very little antitumor activity (Fig. 7B) and, unlike in the AB12 model in BALB/c mice, treatment with PDT did induce strong antitumor activity.

We further utilized the Winn assay in our models to determine whether the addition of TI to PDT directly affected CD8^+^ T-cell activity. In the highly immunogenic AB12 model, very little direct effects on CD8^+^ T-cell activity were seen (Fig. 4B). In contrast, the addition of TI to PDT in the AE17o model markedly blunted the activity of the CD8^+^ T cells (Fig. 7B). The mechanism of this T-cell suppression is under investigation, but may be related to TI-induced stimulation of immunosuppressive factors such as IL6, prostaglandin E2, or catecholamines, all known to be associated with surgically induced immunosuppression (10–15).

To explore possible indirect mechanisms of TI-induced immunosuppression, especially in the AB12 model where very little direct T-cell inhibition was noted, we expanded our Winn assays to include splenocytes. This allowed us to inject the same number of CD8^+^ T cells as above, but now in combination with potentially tumor-induced immunosuppressive cells such as CD4^+^ Tregs and MDSCs existing in the spleen. In both the AB12 and AE17o tumor models, PDT was able to induce strong antitumor activity and the addition of TI to PDT blunted this response (Figs. 5A and 7C). Together, studies of these tumor models show TI/PDT generates less antitumor immunity than PDT in a manner that is transferable via splenocytes. Although we have not definitively proven which suppressor cells were responsible for this TI-induced inhibition, our flow cytometric analysis of the splenic cells suggested that TI/PDT did not change the % of CD4^+^ Tregs, but did increase the fraction of granulocytic MDSC (CD11b^+^/Ly6G^+^), a cell population known to inhibit T-cell function (32, 33).

We posit that transfer of granulocytic MDSCs alongside tumor-directed CD8^+^ T cells that were taken from the spleen limited the overall response in recipient mice in the Winn assay. This conclusion is in line with surgical investigations in various tumor models. Abdominal nephrectomy increased the level of splenic granulocytic MDSCs in mice bearing B16lacZ tumors (34). Although resection of 4T1 tumors was initially shown to reduce MDSCs, the population of MDSCs that remained significantly increased the colonization of 4T1 cells on the lungs versus unresected tumors (35). Treating surgically injured mice with an adenoviral vaccination significantly lowered the long-term immune response compared with mice treated with vaccination alone, driven specifically by granulocytic MDSCs (18). Notably, these authors observed no changes in antigen-specific CD8^+^ T cells, monocytic MDSCs, or Tregs, indicating modifying granulocytic MDSCs was enough to restrict response to the adenoviral vaccine.

IL6 is a pleiotropic cytokine; therefore, it is possible that IL6 released in response to TI may stimulate CD8^+^ T cells or CD4^+^ Th17 cells at the same time it is promoting immunosuppression (36). Our results in the AB12 mesothelioma line suggested that CD8^+^ T cells taken from the spleen of TI/PDT-treated mice retain tumor cytotoxicity when transferred *in vivo* into treatment-naïve tumors. Antitumor immunity was only restricted by TI/PDT when CD8^+^ T cells were cocultured alongside other splenocytes. The result of this pleiotropic effect appears to be a prolongation of the median time to regrowth in tumors treated with TI/PDT compared with TI alone: 16 days in TI/PDT compared with 9.5 days in TI. However, no CRs were observed with TI/PDT compared with the 30% observed in tumors treated with PDT alone.

It is important to recognize the limitations of our studies. Only mesothelioma tumor models were studied, although of different subtypes in different mouse strains. We could not utilize orthotopic models of mesothelioma, which grow tumors inside the intact ribcage, due to the need to incise or debulk tumors in survival surgeries. Tumor incision is a model of surgical injury but does not exactly mimic the situation of intrathoracic cytoreduction surgery. However, our previous work demonstrated that the release of cytokines in response to incising mesothelioma tumors on the flanks of mice is comparable with that of patients with mesothelioma who have undergone surgical debulking (19). For technical reasons, our work used splenic T cells and immunosuppressive cells rather than cells from the tumor microenvironment. Despite these limitations, the current work establishes the immunosuppressive effects of surgery on subsequent PDT and provides direction to further study the cell types responsible for this phenotype.

In summary, PDT significantly improves long-term response to surgical cytoreduction, but does so in competition with an immunosuppressive effect of surgical injury that blunts the antitumor effects of PDT. We found this to be mediated by multiple mechanisms. In one of our models (AE17o), this includes a direct effect on CD8^+^ T-cell antitumor activity. In both models, surgery appears to stimulate increases in splenic suppressor cell activity. The processes through which surgery causes these changes are not yet known, but are likely accompanied by surgically induced increases in immunosuppressive inhibitory factors such as IL6, cortisol, TGFβ, IL10, VEGF, and/or PGE2 (10–15). Indeed, our mesothelioma surgery/PDT trial reveals high circulating IL6 levels at the conclusion of macroscopic complete resection (19) and was reproduced in mice using the TI model (Supplementary Fig. S2A). Inhibition of IL6 (or the IL6 receptor) or PGE2 introduced in the perioperative period could take advantage of the benefits of cytoreduction surgery while minimizing the immunosuppressive effects.

## Supplementary Material

Supplementary Figure 1 Supplemental Figure 1. Growth Curves of Individual Mice Treated With Surgical Resection, Photodynamic Therapy, or Tumor Incisions

Supplementary Figure 2 Supplemental Figure 2. TI promotes acute inflammation

Supplementary Figure 3 Supplemental Figure 3. Preceding PDT with TI significantly reduces response in male AB12-bearing mice

Supplementary Figure 4 Supplemental Figure 4. PDT response is not altered when it is followed by TI

Supplementary Figure 5 Supplemental Figure 5. Gating Scheme for Splenocyte Immunophenotyping
