# Supplementary material for: Surgical Inflammation Alters Immune Response to Intraoperative Photodynamic Therapy
Source: Cancer Res Commun. 2023 Sep 11;3(9):1810–22. doi: 10.1158/2767-9764.CRC-22-0494 (PMC10494787; doi:10.1158/2767-9764.CRC-22-0494)
Supplement: Supplementary Figure 1 — Supplemental Figure 1. Growth Curves of Individual Mice Treated With Surgical Resection, Photodynamic Therapy, or Tumor Incisions [file crc-22-0494-s01.pdf]

**Supplemental Figure 1. Growth Curves of Individual Mice Treated With Surgical Resection, Photodynamic Therapy, or Tumor Incisions.** Tumor regrowth curves depicting the timing of tumor growth to the 400 $mm^3$ endpoint, at which point tumors were censored in Kaplan Meier curves in Figure 1. The proportion of complete responses are depicted as the horizontal asymptote of Kaplan Meier curves in Figure 1.

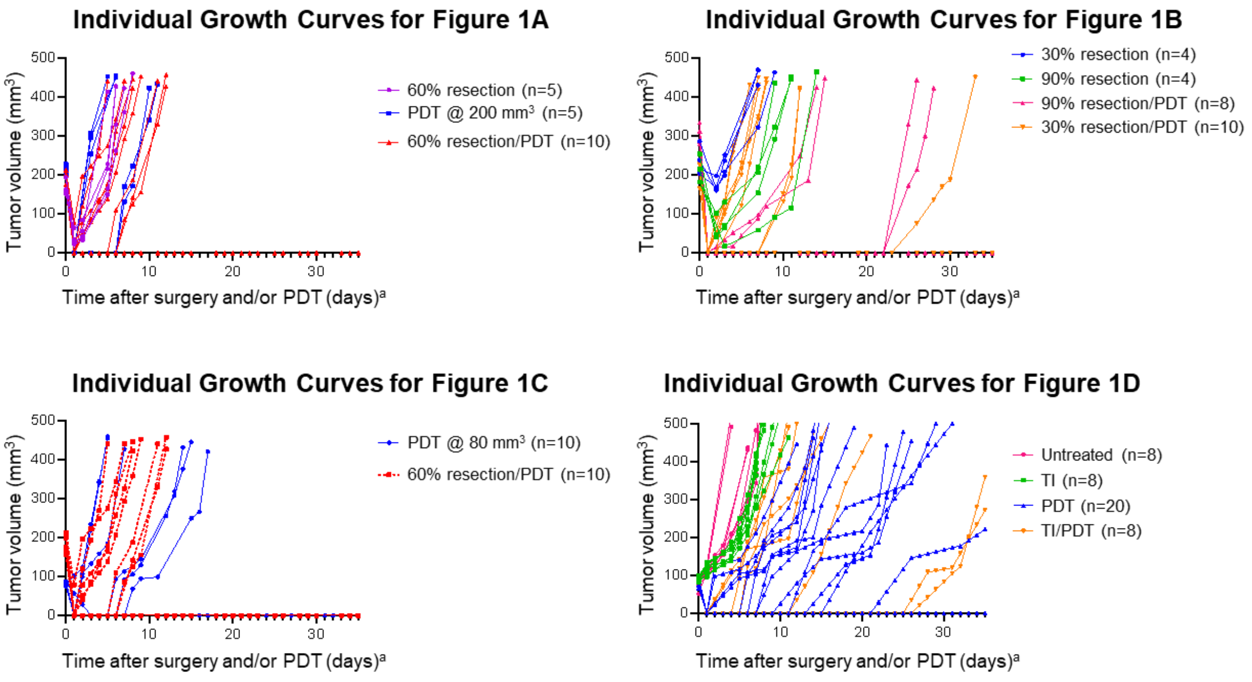


[a]No tumor regrowth was observed for any mice at day 35 through 90.
