# Supplementary material for: Surgical Inflammation Alters Immune Response to Intraoperative Photodynamic Therapy
Source: Cancer Res Commun. 2023 Sep 11;3(9):1810–22. doi: 10.1158/2767-9764.CRC-22-0494 (PMC10494787; doi:10.1158/2767-9764.CRC-22-0494)

Supplemental Figure 2. TI promotes acute inflammation. (A) ELISA was employed to analyze the level of IL-6 in tumor tissue after TI administration. TI induced an increase in mean IL-6 4 hours after exposure to TI. (**B**) Gating strategy for analysis of CD11b$^+$ Ly6G$^+$ granulocytes. (**C**) TI increased the mean level of tumor-infiltrating CD11b$^+$ Ly6G$^+$ granulocytes 4 hours after administration and remained elevated at 19 hours after administration compared to control tumors. n=14 for control; n=3 for skin; n=4-6 for TI-treated time points. *P<0.05,**P<0.01,***P<0.001

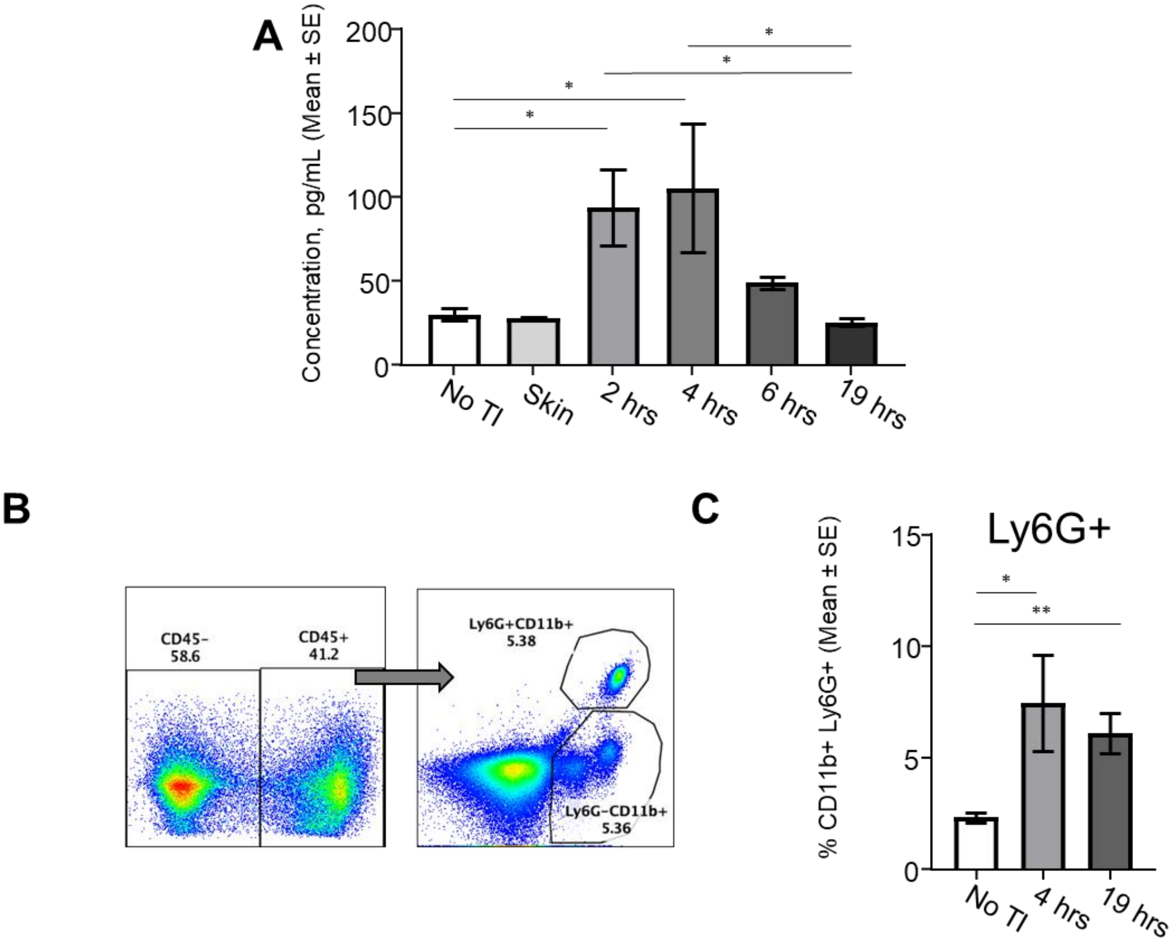

Supplement: Supplementary Figure 2 — Supplemental Figure 2. TI promotes acute inflammation [file crc-22-0494-s02.pdf]
