# Supplementary material for: Surgical Inflammation Alters Immune Response to Intraoperative Photodynamic Therapy
Source: Cancer Res Commun. 2023 Sep 11;3(9):1810–22. doi: 10.1158/2767-9764.CRC-22-0494 (PMC10494787; doi:10.1158/2767-9764.CRC-22-0494)

Supplemental Figure 3. Preceding PDT with TI significantly reduces response in male AB12-bearing mice. Male AB12 mice were treated with PDT in the presence or absence of TI. As in female mice, the absence of TI significantly increased the response rate of PDT-treated mice (P=0.0396). Untreated, n=10; TI, n=10; PDT, n=15; TI/PDT, n=15.

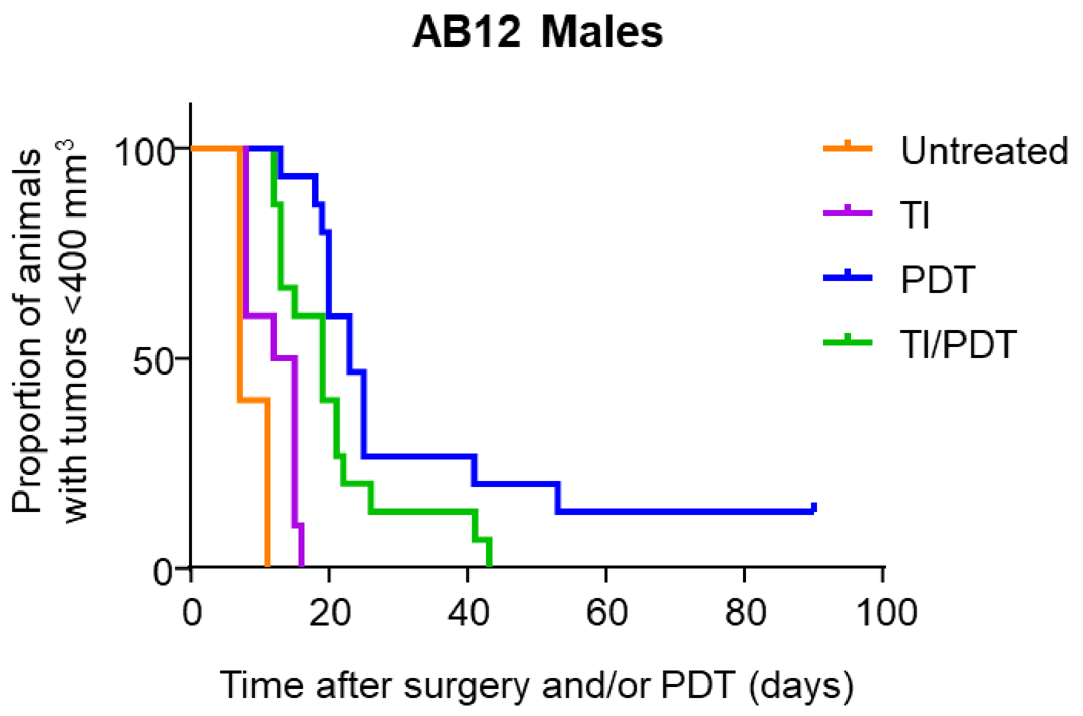

Supplement: Supplementary Figure 3 — Supplemental Figure 3. Preceding PDT with TI significantly reduces response in male AB12-bearing mice [file crc-22-0494-s03.pdf]
