# Supplementary material for: Surgical Inflammation Alters Immune Response to Intraoperative Photodynamic Therapy
Source: Cancer Res Commun. 2023 Sep 11;3(9):1810–22. doi: 10.1158/2767-9764.CRC-22-0494 (PMC10494787; doi:10.1158/2767-9764.CRC-22-0494)

Supplemental Figure 4. PDT response is not altered when it is followed by TI. To determine the effect of the timing of TI administration on PDT-induced response, tumors were treated with PDT immediately prior to TI exposure (PDT/TI). No difference in response rate was observed compared to mice treated with PDT alone (*P*=0.8891). PDT, n=20; PDT/TI, n=23. Plots in dotted lines are replotted from previous figures to provide comparison

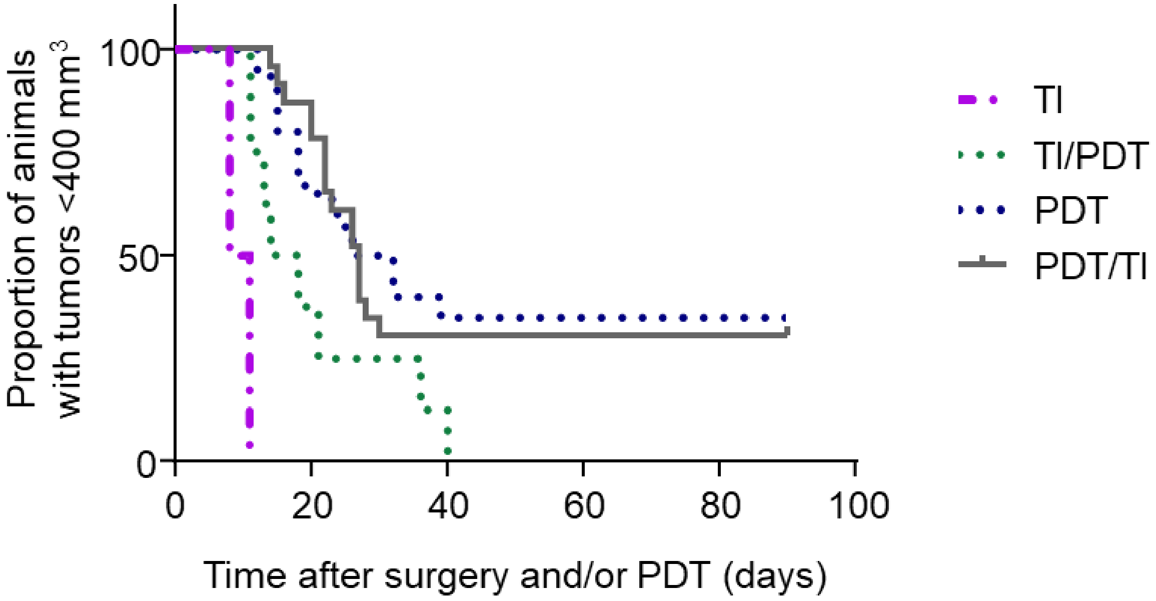

Supplement: Supplementary Figure 4 — Supplemental Figure 4. PDT response is not altered when it is followed by TI [file crc-22-0494-s04.pdf]
