# Supplementary material for: Surgical Inflammation Alters Immune Response to Intraoperative Photodynamic Therapy
Source: Cancer Res Commun. 2023 Sep 11;3(9):1810–22. doi: 10.1158/2767-9764.CRC-22-0494 (PMC10494787; doi:10.1158/2767-9764.CRC-22-0494)

**Supplemental Figure 5. Gating Scheme for Splenocyte Immunophenotyping.** Cells were selected based on size and granularity and doublets were excluded. To identify CD4+ and CD8+ lymphocytes, cells were selected based on viability and CD3 expression and were then gated based on expression of either CD4 or CD8. To identify neutrophils and/or myeloid-derived suppressor cells (MDSCs), cells were selected based on viability and then gated according to expression of CD11b and Ly6G (CD11b+/Ly6G+: neutrophils; CD11b+Ly6G-: MDSCs).

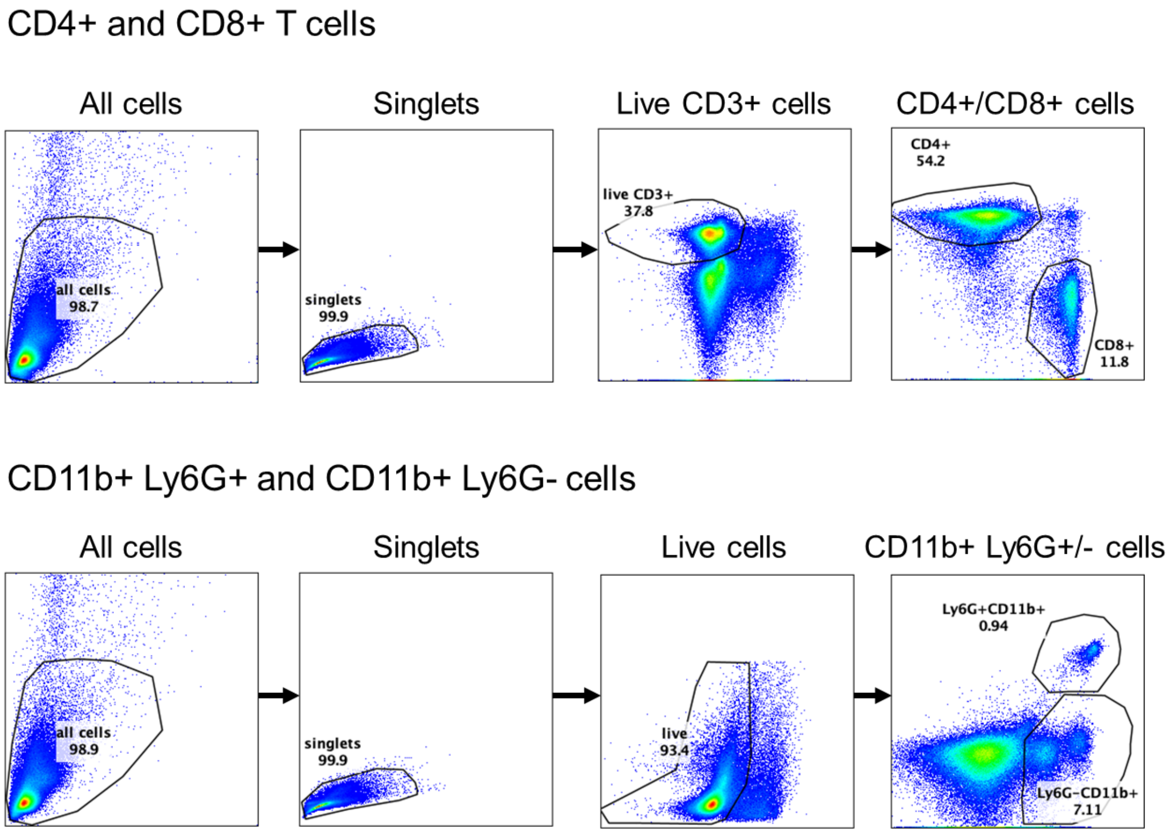

Supplement: Supplementary Figure 5 — Supplemental Figure 5. Gating Scheme for Splenocyte Immunophenotyping [file crc-22-0494-s05.pdf]
